# 163. High Prevalence of Urogenital and Rectal *Mycoplasma genitalium* in U.S. MSM with a History of STIs in the Last Year

**DOI:** 10.1093/ofid/ofab466.163

**Published:** 2021-12-04

**Authors:** Emma D Bainbridge, Olusegun O Soge, Cole Grabow, Stephanie Cohen, Julia C Dombrowski, Jade Fairbanks, Connie Celum, Annie Luetkemeyer

**Affiliations:** 1 University of California San Francisco, San Francisco, California; 2 University of Washington, Seattle, Washington; 3 UCSF, San Francisco, California

## Abstract

**Background:**

*M. genitalium* (*M. gen*) is an under-recognized sexually transmitted bacterial pathogen that causes 15-25% of nongonococcal urethritis (NGU) in men. Asymptomatic *M. gen* may serve as a reservoir, lead to transmission to sexual contacts, and drive the development of drug resistance. *M. gen* may be associated with an increased risk of HIV acquisition, as seen in some studies. Data are limited on *M. gen* prevalence among U.S. men who have sex with men (MSM) living with HIV or HIV-uninfected and on pre-exposure prophylaxis (PrEP).

**Methods:**

We analyzed baseline prevalence of urogenital and rectal *M. gen* using the Aptima *Mycoplasma genitalium* nucleic acid amplification test in participants enrolled in DoxyPEP, an ongoing randomized, open label trial of the effectiveness of doxycycline post-exposure prophylaxis (PEP) on incidence of gonorrhea, chlamydia, and early syphilis among MSM and transgender women living with HIV or on PrEP in San Francisco and Seattle (NCT03980223). Participants completing at least one follow up visit were also assessed for *M. gen* persistence, clearance, and incidence. Testing was at regular intervals and not symptom driven.

**Results:**

This analysis included 122 men; 34% with HIV and 66% on PrEP. In the prior 12 months, 18.9% had a diagnosis of syphilis, 58.2% chlamydia, and 63.9% gonorrhea. At baseline, *M. gen* was present in at least one site in 24%; 9% in the urine and 16% in the rectum, with 1 testing positive at both sites. *M. gen* presence was not associated with age, ethnicity, race, HIV status, number of partners in the past 3 months, or bacterial STI in the past 3 months. 65 participants had follow up tests a median of 9.1 months after baseline (IQR 7.8-9.8); among 7 participants with urogenital *M. gen* at baseline, *M. gen* cleared in 6 and persisted in 1. Among 11 participants with rectal *M. gen* at baseline, *M. gen* cleared in 4 cleared and persisted in 7. At follow up, *M. gen* was detected in 2 urine and 9 rectal specimens in those previously negative at these sites.

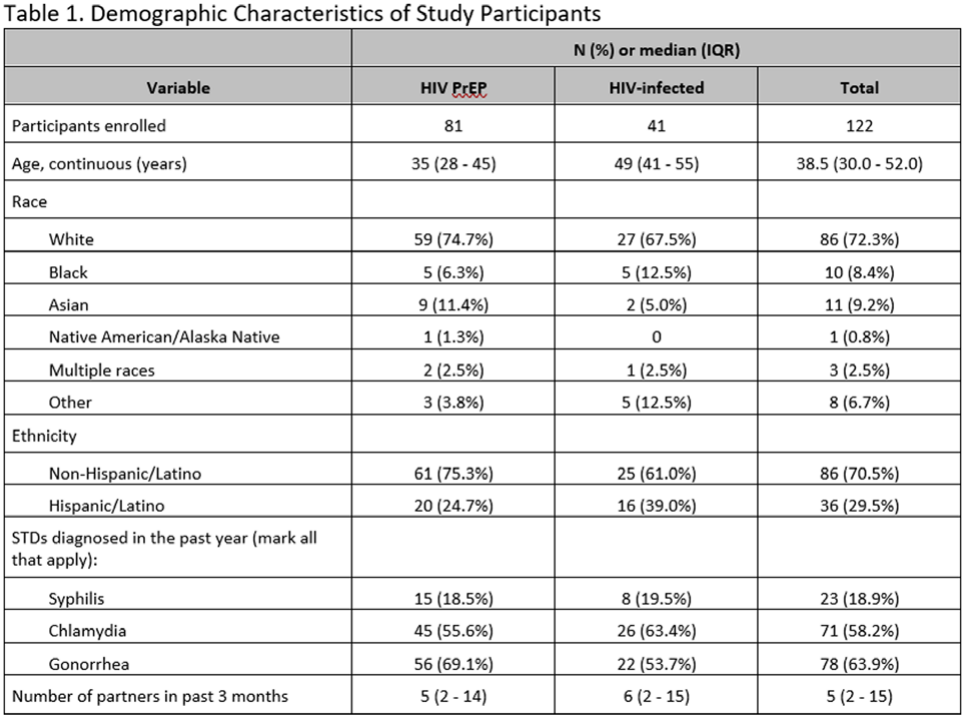

Figure 1. Baseline prevalence of urogenital and rectal M. genitalium in MSM at high risk for STIs enrolled in DoxyPEP

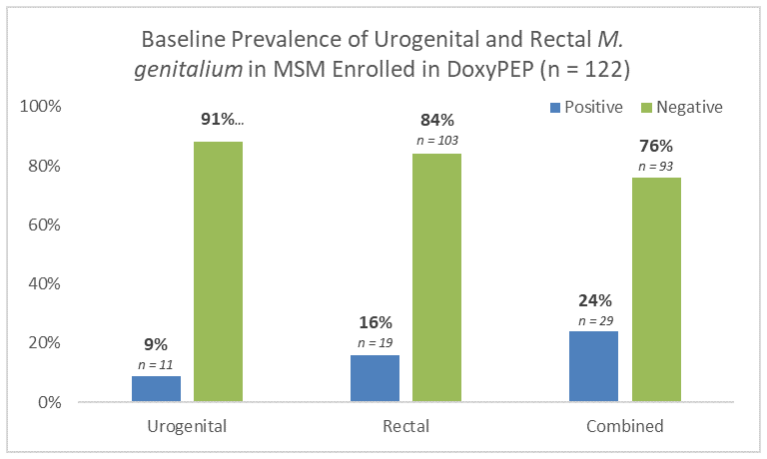

Figure 2. Detection of urogenital and rectal M. genitalium among participants with baseline and follow up testing

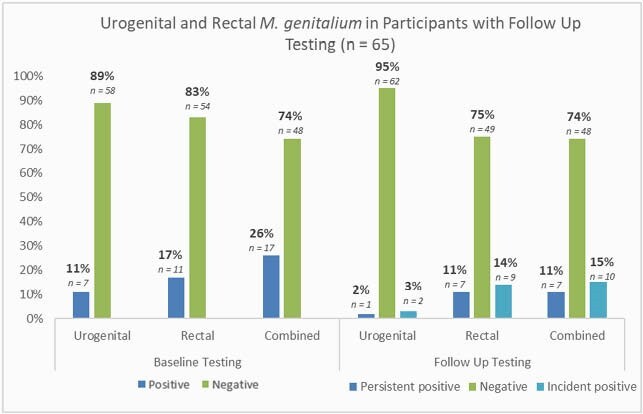

**Conclusion:**

In this cohort of MSM with a recent diagnosis of a bacterial STI, routine testing identified urogenital or rectal *M. gen* in 24% of participants at baseline and 31% at either baseline or follow-up. The association of persistent *M. gen* with the risk for subsequent symptomatic infection and drug resistance merits further investigation.

**Disclosures:**

**Emma D. Bainbridge, MD, MPH**, **Hologic** (Grant/Research Support) **Olusegun O. Soge, PhD**, **Hologic Inc.** (Grant/Research Support)**SpeeDx Inc.** (Grant/Research Support) **Annie Luetkemeyer, MD**, **Cepheid** (Grant/Research Support)**Hologic** (Grant/Research Support)**Mayne Pharma** (Grant/Research Support)

